# Oxytocin: A Shield against Radiation-Induced Lung Injury in Rats

**DOI:** 10.3390/tomography10090101

**Published:** 2024-08-29

**Authors:** Ahmet Kayalı, Duygu Burcu Arda, Ejder Saylav Bora, Yiğit Uyanikgil, Özüm Atasoy, Oytun Erbaş

**Affiliations:** 1 Department of Emergency Medicine, Faculty of Medicine, Izmir Katip Çelebi University, 35620 Izmir, Türkiye; ejdersaylav.bora@ikc.edu.tr; 2 Department of Pediatrics, Istanbul Taksim Research and Training Hospital, 34433 Istanbul, Türkiye; dr.duyguburcu@hotmail.com; 3 Department of Histology and Embryology, Faculty of Medicine, Ege University, 35030 Izmir, Türkiye; yigit.uyanikgil@ege.edu.tr; 4 Department of Radiation Oncology, Giresun Training and Research Hospital, 28100 Giresun, Türkiye; ozumatasoy@gmail.com; 5 Department of Physiology, Faculty of Medicine, Demiroğlu Bilim University, 34394 Istanbul, Türkiye; oytunerbas2012@gmail.com

**Keywords:** radiation pneumonitis, oxytocin, radiotherapy, inflammation

## Abstract

Background: Radiation-induced lung injury (RILI), a serious side effect of thoracic radiotherapy, can lead to acute radiation pneumonitis (RP) and chronic pulmonary fibrosis (PF). Despite various interventions, no effective protocol exists to prevent pneumonitis. Oxytocin (OT), known for its anti-inflammatory, antiapoptotic, and antioxidant properties, has not been explored for its potential in mitigating RILI. Materials and Methods: This study involved 24 female Wistar albino rats, divided into three groups: control group, radiation (RAD) + saline, and RAD + OT. The RAD groups received 18 Gy of whole-thorax irradiation. The RAD + OT group was treated with OT (0.1 mg/kg/day) intraperitoneally for 16 weeks. Computerizing tomography (CT) imaging and histopathological, biochemical, and blood gas analyses were performed to assess lung tissue damage and inflammation. Results: Histopathological examination showed significant reduction in alveolar wall thickening, inflammation, and vascular changes in the RAD + OT group compared to the RAD + saline group. Biochemical analysis revealed decreased levels of TGF-beta, VEGF, and PDGF, and increased BMP-7 and prostacyclin in the RAD + oxytocin group (*p* < 0.05). Morphometric analysis indicated significant reductions in fibrosis, edema, and immune cell infiltration. CT imaging demonstrated near-normal lung parenchyma density in the RAD + oxytocin group (*p* < 0.001). Conclusion: Oxytocin administration significantly mitigates radiation-induced pneumonitis in rats, implying that is has potential as a therapeutic agent for preventing and treating RILI.

## 1. Introduction

Radiotherapy is crucial in treating localized primary malignancies that affect the chest wall or intrathoracic malignancies [1]. Radiation-induced lung injury (RILI) is a consequence of the additional effects of radiotherapy on the lung. The progression of lung damage caused by radiation varies from acute radiation pneumonitis (RP) to chronic pulmonary fibrosis (PF) [2].

Pneumonitis is the commonly used term for the inflammation of lung tissue. It is a term for chronic lung inflammation caused by non-infectious factors such as irritation, radiation, aspiration, and drug-induced inflammation [1,2]. Radiation pneumonitis is diagnosed clinically by considering the patient’s history of radiation exposure, analyzing imaging results, and identifying typical symptoms. Other potential causes, such as infection, pulmonary embolism (PE), heart disease, drug-induced pneumonitis, and tumor progression, must be ruled out [1]. 

The root causes of RILI are diverse, encompassing genetic alterations, the impact of signaling pathways, the merging of different cells, and the production of mediators like cytokines and chemokines [3]. Severe RP and PF can significantly diminish the quality of life despite the effectiveness of the anti-tumor treatment in patients [4]. Pneumonias developing on the background of BOOP syndrome [5], acute respiratory distress syndrome [6], and most commonly, cryptogenic organizing pneumonia (COP) [7] are serious diseases that can be life-threatening after treatment. Therefore, acting before pneumonitis develops or treating pneumonitis early is very important and lifesaving. Despite all molecular and pharmacological studies [8,9,10], no protocol exists to prevent pneumonitis.

Oxytocin (OT), a hormone produced in the paraventricular and supraoptic nuclei of the hypothalamus, has been found to possess anti-inflammatory properties [11]. OT plays a role in uterine contraction during childbirth and the milk-ejection reflex during breastfeeding [11]. It also has positive effects in reducing anxiety and stress disorders [12], as well as in preventing damage caused by lack of blood flow and subsequent restoration of blood flow to the liver [13], heart, myocardium [14], kidneys [15], and brain [16]. Due to its anti-inflammatory, antiapoptotic, and antioxidant properties, OT is believed to be a beneficial substance for reducing ischemia/reperfusion injury [17,18]. Nevertheless, its contribution to RILI has yet to be examined.

The severity of lung injuries caused by radiation depends on several factors, including the total dose of radiation, the volume of tissue exposed to radiation, and whether radiotherapy is used in conjunction with surgery or chemotherapy [19]. These factors can be regarded as constraints in chest radiotherapy; therefore, they should be considered to prevent or reduce lung injuries. While reducing the volume of irradiation or the total radiation dose may be seen as the most effective approach to alleviate RILI, it could decrease therapeutic effectiveness [20,21,22]. Moreover, OT’s contribution to RILI has yet to be examined. 

Unfortunately, the risk of exposure to nuclear fallout is high in our world, where there are still nuclear power plants and threats of nuclear war. The exposure to fallout by inhalation shows us that our current study is not limited to the complications of radiation therapy and that acute treatments related to pre- and post-exposure should be known. In this study, we investigated the effect of OT administration in radiation pneumonitis and the mechanism by which the OT effect may occur. 

## 2. Materials and Methods

### 2.1. Animal

For this study, twenty-four female Wistar albino rats, weighing about 150–200 g and aged 10–12 weeks, were used in the study. The experiments carried out in this study adhered to the rules specified in the Guide for the Care and Use of Laboratory Animals. The rats used in the experiment were obtained from the Experimental Animal Laboratory of Science University. The rats were given unrestricted access to food and housed in steel cages inside a controlled environment, maintained at a temperature of 22 ± 2 °C, and subjected to a 12 h light/dark cycle.

### 2.2. Experimental Protocol

Twenty-four male Wistar rats were taken to study. For the radiation injury model, 16 rats received whole-thorax irradiation (RAD). Rats given whole-thorax irradiation were randomly divided into two groups.

Study groups were designed as follows:

Group 1: Normal control (orally fed control, *n* = 8); 

Group 2 (RAD + saline, *n* = 8): Thorax irradiation and 1 mL/kg/day % 0.9 NaCl saline group via intraperitoneally (i.p); 

Group 3 (RAD + OT, *n* = 8): Thorax irradiation and OT 0.1 mg/kg/day (OT 10 U/mL, Vetas) via i.p. 

All treatments were daily and given following RAD admission for 16 weeks. We conducted thorax CT imaging on all animals under ketamine anesthesia 16 weeks later. After the trial, animals were slaughtered (cervical dislocation) under ketamine (100 mg/kg, Richterpharma AG, Wels, Austria)/xylazine (50 mg/kg, Rompun, Bayer, Leverkusen, Germany) anesthesia. Blood samples were obtained for biochemical examination via cardiac puncture. 

### 2.3. Radiotherapy

All irradiations were performed while the subjects were under anesthesia induced by a combination of ketamine (50 mg/kg) and xylazine (10 mg/kg). The animals’ limbs were taped, and they were placed in a supine position on a Plexiglas plate. A 6 MV X-ray linear accelerator system manufactured by Varian Medical Systems in Palo Alto, San Jones, CA, USA, was used to administer a single dosage of 18 Gy of X-radiation to the thoracic area of rats. The radiation was supplied at a source to surface distance of 100 cm and required 600 monitor units. 

CT images of the rat lung tissue determined by the radiation oncologist and dose prescribed as the whole thorax received 98% of the prescribed dose.

### 2.4. CT Examination of the Lung

The examinations were conducted using a 16-slice multi-detector row CT scanner (Somatom Go Now, Siemens Healthcare, Erlangen, Germany) while the patient was lying down. No contrast media were used, but an anesthetic agent was injected. The rats were administered a combination of ketamine (50 mg/kg, Ketasol, Richterpharma AG, Wels, Austria) and xylazine (10 mg/kg, Rompun, Bayer, Leverkusen, Germany) intraperitoneally to induce deep anesthesia. All animals were securely immobilized on the scanning table using appropriate materials to prevent any movement artifacts. The study used scanning parameters of 120 kV, variable mAs determined by the automatic exposure control system, and a slice thickness of 1 mm. The scanning range encompassed the C3 vertebrae to the diaphragm, covering both the apex and base of the lung. The CT examination for each rat necessitated an imaging duration ranging from 0.8 to 1 s. Once the pictures were obtained, they were rebuilt with a slice thickness of 1 mm, using a matrix size of 512 × 512 and a sharp reconstruction kernel known as KernelBr64. Three radiologists (I.H.S, B.O, and S.G.G) assessed all pictures without knowledge of the animals’ test results and groups. Six equally sized areas of interest (ROI), each measuring 2.153 mm^2^, were placed on axial images with a parenchymal window at the heart apex level. The regions of interest (ROIs) were uniformly distributed throughout the upper, middle, and lower zones of both lungs, with two ROIs located in each zone. Measures were implemented to prevent the placement of the ROI in close proximity to major blood arteries, airways, and bones.

### 2.5. Arterial Blood Gas Analysis

After the operation, blood samples (0.2 mL) were taken from the carotid arteries of rats in each group. A blood gas analyzer tested These samples for PaO_2_ and PaCO_2_.

### 2.6. Histopathological Examination of the Lung

To conduct histological investigation, rats were anesthetized intraperitoneally with ketamine (100 mg/kg, Alfamine^®^, Alfasan International BV, A Woerden, Holland) and xylazine (50 mg/kg, Alfazyne^®^, Alfasan International BV, A Woerden, Holland). Then, 200 mL of 4% formaldehyde solution in 0.1 M phosphate-buffered saline was perfused. The lung tissues that were chosen at random were placed in paraffin before being examined histologically. Twenty-five distinct sections were extracted from five blocks acquired from each lung tissue block and subjected to staining. Twenty-five distinct regions were assessed in five lung sections chosen at random. Lung sections treated with formalin and fixed were stained using the hematoxylin and eosin (H&E) method. The sections were five μm thick. The Olympus C-5050 digital camera (Olympus Inc., Tokyo, Japan) photographed all sections, mounted on the Olympus BX51 microscope (Olympus Inc., Tokyo, Japan). Two independent blind histologists conducted a lung histopathological evaluation. The histopathological lung damage was evaluated based on the extent of fibrosis, edema, and infiltration of immune cells.

### 2.7. Lung Tissue Biochemical Analysis 

Following the sacrifice, the lungs were promptly extracted and stored at a temperature of −20 °C until they could be subjected to biochemical analysis. To perform tissue analysis, the tissues were broken down using a glass homogenizer in a solution consisting of 5 times the volume of phosphate-buffered saline (pH, 7.4). The resulting mixture was then subjected to centrifugation at a force of 5000× *g* for a duration of 15 min. Next, the liquid portion containing the suspended particles was gathered, and the overall amount of protein in the tissue samples was measured using Bradford’s technique, which involves using bovine serum albumin as a reference [23]. 

The levels of transforming growth factor-beta (TGF-beta), bone morphogenetic protein-7 (BMP-7), vascular endothelial growth factor (VEGF), platelet-derived growth factor (PDGF), and prostacycline in the tissue supernatants were measured using readily accessible rat enzyme-linked immunosorbent assay (ELISA) kits from Sigma Aldrich in St. Louis, St. Louis, Missouri, USA. The samples of each animal were measured twice, in accordance with the parameters provided by the manufacturer. The absorbances were quantified using a reader for micro plates (MultiscanGo, Thermo Fisher Scientific Laboratory Equipment, Portsmouth, NH, USA).

### 2.8. Statistical Analysis 

The data in the study were analyzed using the IBM SPSS Statistics Standard Concurrent User V 26 (IBM Corp., Armonk, New York, NY, USA) statistical software package. The standard error of the mean (SEM) is represented as mean ± in descriptive statistics for parameters. The one-way analysis of variance (ANOVA) test was employed for variables with more than two groups. If the analysis of variance (ANOVA) result was statistically significant, the Bonferroni test was used as a multiple comparison test. A *p*-value of less than 0.001 was deemed to be highly statistically significant.

## 3. Results

### 3.1. Histopathological Findings

**Figure 1:**
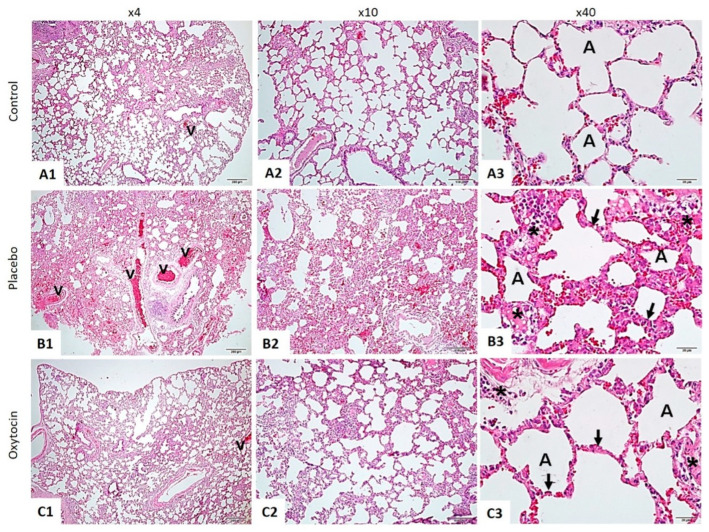
The lung tissue was stained using the hematoxylin and eosin method. In the Control Group (**A1**–**A3**), the lung tissue displayed a normal structure, with intact alveoli (A) and blood vessels (v). This contrasted sharply with the RAD + Saline Group (**B1**–**B3**), where significant thickening of the alveolar walls (indicated by arrows), increased inflammation (marked by asterisks), and pronounced vascular changes (v) were observed. Notably, the RAD + Oxytocin Group (**C1**–**C3**) showed considerable improvement, with reduced thickening of the alveolar walls (arrows), less inflammation (asterisks), and more normalized vascular structures (v). In the control group (**A1**–**A3**), the lung tissue displayed a standard structure, with intact alveoli (A) and blood vessels (v). This contrasted sharply with the RAD + saline group (**B1**–**B3**), where significant thickening of the alveolar walls (indicated by arrows), increased inflammation (marked by asterisks), and pronounced vascular changes (v) were observed. Notably, the RAD + OT group (**C1**–**C3**) showed considerable improvement, with reduced thickening of the alveolar walls (arrows), less inflammation (asterisks), and more normalized vascular structures (v).

The histopathological examination of lung tissues stained with hematoxylin and eosin (H&E) revealed marked differences between the three groups (Figure 1).

### 3.2. Biochemical Analysis of Lung Tissue

The biochemical analysis of lung tissue demonstrated significant differences across the groups in several key proteins and mediators (Table 1).

TGF-beta: The RAD + saline group exhibited significantly elevated levels of TGF-beta (1.46 ± 0.1 pg/mg protein) compared to the control group (0.85 ± 0.04 pg/mg protein, * *p* < 0.01). Treatment with OT significantly reduced TGF-beta levels to 1.05 ± 0.06 pg/mg protein (# *p* < 0.05 vs. RAD + saline group).

BMP-7: Levels of BMP-7 were significantly lower in the RAD + saline group (0.33 ± 0.08 pg/mg protein) compared to the control group (0.45 ± 0.02 pg/mg protein, ** *p* < 0.001). However, OT treatment elevated BMP-7 levels to 0.58 ± 0.03 pg/mg protein (## *p* < 0.001 vs. RAD + saline group).

VEGF: VEGF levels were notably higher in the RAD + saline group (12.5 ± 3.7 pg/mg protein) compared to the control group (6.2 ± 0.4 pg/mg protein, ** *p* < 0.001). OT administration reduced VEGF levels to 8.9 ± 2.5 pg/mg protein (## *p* < 0.001 vs. RAD + saline group).

PDGF: The RAD + saline group showed significantly increased PDGF levels (1.22 ± 0.09 pg/mg protein) relative to the control group (0.38 ± 0.01 pg/mg protein, * *p* < 0.01). OT treatment decreased PDGF levels to 0.71 ± 0.6 pg/mg protein (# *p* < 0.05 vs. RAD + saline group).

Prostacyclin: The RAD + saline group had lower prostacyclin levels (64.5 ± 6.6 pg/mg protein) compared to the control group (108.2 ± 15.1 pg/mg protein, * *p* < 0.01). With OT treatment, prostacyclin levels increased to 85.3 ± 7.05 pg/mg protein (# *p* < 0.05 vs. RAD + saline group).

### 3.3. Morphometric Analysis

Morphometric analysis highlighted significant differences in the percentages of fibrosis, edema, and immune cell infiltration among the groups (Table 2).

Fibrosis: The percentage of fibrosis was substantially higher in the RAD + saline group (45.2 ± 6.5%) compared to the control group (1.5 ± 0.8%, * *p* < 0.0001). OT treatment reduced fibrosis to 18.6 ± 4.9% (# *p* < 0.001 vs. RAD + saline group).

Edema: The RAD + saline group exhibited a higher percentage of edema (32.1 ± 7.2%) compared to the control group (2.3 ± 1.1%, * *p* < 0.0001). OT administration significantly decreased edema to 10.7 ± 4.1% (# *p* < 0.001 vs. RAD + saline group).

Immune cell infiltration: Immune cell infiltration was markedly increased in the RAD + saline group (28.5 ± 5.4%) compared to the control group (6.4 ± 2.5%, * *p* < 0.0001). Treatment with OT reduced infiltration to 15.5 ± 7.8% (# *p* < 0.001 vs. RAD + saline group).

### 3.4. Computed Tomography (CT) Analysis

**Figure 2:**
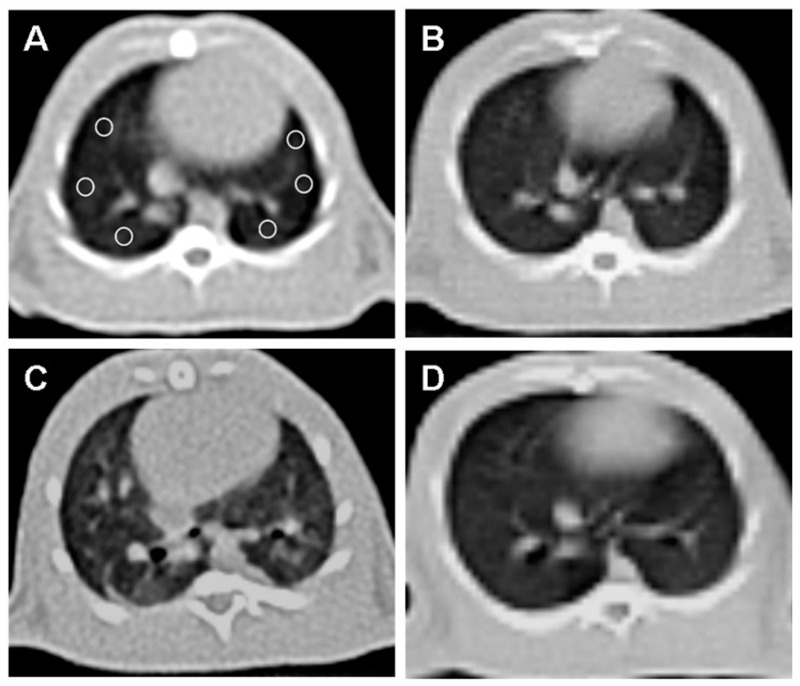
Axial non-contrast computed tomography (CT) image of the lung at the level of the heart, with six equally sized regions of interest (ROI) placed (**A**). (**A**) and (**B**) CT image of the rat’s lungs in the control group without abnormalities. (**C**) The irradiation group of rats showed a notable rise in lung density and fibrosis compared to the normal group. (**D**) The lung image of the rat that received OT treatment showed that the lung tissue density was similar to that of the normal group.

CT imaging provided further evidence of the beneficial effects of OT treatment (Figure 2).

Normal lung parenchyma was observed in the control group (B). The RAD + saline group (C) showed a significant increase in lung density and fibrosis compared to the control group.

The RAD + OT group (D) displayed lung parenchyma density close to normal, indicating reduced fibrosis and structural damage. A quantitative CT analysis of the Hounsfield unit (HU) values revealed significant differences:

Control group: 618.5 ± 9.5 HU.

RAD + saline group: 465.1 ± 8.8 HU (* *p* < 0.0001 vs. control group).

RAD + OT group: 594.2 ± 10.7 HU (# *p* < 0.001 vs. RAD + saline group).

### 3.5. Blood Gas Analysis

Blood gas measurements showed significant differences in arterial oxygen (PaO_2_) and carbon dioxide (PaCO_2_) levels (Table 3).

PaO_2_: The RAD + saline group had lower PaO_2_ levels (90.2 ± 3.8 mmHg) compared to the control group (102.1 ± 8.2 mmHg, * *p* < 0.05). OT treatment improved PaO_2_ to 98.1 ± 6.5 mmHg (# *p* < 0.05 vs. RAD + saline group).

PaCO_2_*:* PaCO_2_ levels were higher in the RAD + saline group (51.1 ± 6.5 mmHg) compared to the control group (36.3 ± 2.9 mmHg, * *p* < 0.05). OT treatment reduced PaCO_2_ to 44.3 ± 5.9 mmHg (# *p* < 0.05 vs. RAD + saline group).

## 4. Discussion

During radiotherapy for tumors in the thorax, the lungs are unavoidably exposed to radiation, because they are radiosensitive organs [20,24]. The sole circumstance that can eradicate the complexities of irradiation is a reduction in dosage. Pneumonitis is a potentially fatal side effect resulting from medical intervention. The primary objective of current research is to gain a more comprehensive understanding of the pathophysiology of pneumonitis and to assess the viability of utilizing OT as a preventive measure against early complications caused by radiation.

Lung injuries caused by ionizing radiation are examined in both the acute and late phases. During the initial stage, exudation, alveolar edema, alveolar septal thickening with infiltration of mononuclear cells, and vascular congestion can be observed. These symptoms are indicative of radiation pneumonitis. These pulmonary injuries with a sudden onset may manifest around one month following radiotherapy [25,26,27]. According to histopathological findings, extensive damage to the alveoli is considered the initial indication of lung injury at this stage [27]. In radiation, pneumoconiosis, hypoxia, and hypocarbia occur, just as in interstitial lung diseases. In the context of blood gas, it may mimic the pulmonary embolism clinic. The problem in pneumonitis is related to ventilation [27]. Since CO_2_ can diffuse faster than O_2_ when the air–blood barrier is disrupted by radiation, oxygen is most affected by this situation, and hypoxia occurs [26]. At the same time, this study correlated with blood gas and tomography results. Thorax CT imaging showed viral-like pneumonia after radiation therapy, whereas the OT-treated group was close to the normal group. In this study, OT treatment given after radiation exposure reversed the pulmonary effects of radiation therapy in a histopathological manner. This demonstrated that inflammatory processes were suppressed, and that OT strongly prevented possible radiation pneumonitis. 

In a study conducted by Sever et al., the authors state that OT demonstrates anti-inflammatory, antioxidant, and protective properties in a model of acute lung injury induced by sepsis, reducing lung damage and inflammation [28]. Moreover, Lin et al. describe that prior administration of OT can decrease the ischemic, inflammatory, and oxidative reactions in rats with heat-induced acute lung injury, which may enhance their chances of survival [29]. In an acute lung injury model induced by sepsis, OT can potentially mitigate inflammatory reactions in mice with LPS-induced acute lung injury, thus protecting against this condition [30]. As we can observe from these previous studies, which were conducted using OT, and from this study, OT behaves similarly by suppressing the inflammation and oxidative stress in radiation-induced lungs. 

TGF-beta has a two-fold impact on lung injury. On the one hand, it improves the removal of microbes [31]. On the other hand, it exacerbates lung damage through different mechanisms, such as increasing the permeability of the epithelial layer and suppressing crucial lung proteins [32,33]. Studies have indicated that ionizing radiation can directly trigger the TGF-β signaling pathway. This is supported by previous reports that show an increase in TGF-β expression after radiation exposure, which is considered a predictor of fibrosis [34]. Inhibiting TGF-beta or its activation could be a promising therapeutic approach to reducing lung injury. [35]. OT is known to be a treatment option for inflammatory diseases [36]. On the other hand, an in vitro study by Mittaud et al. found that neurons can regulate the expression of OT receptors in cultured astrocytes. This regulation can be achieved by stimulating the receptors through the release of TGF or by inhibiting them through specific membrane components whose identity is currently unknown [37]. Several studies have demonstrated that the upregulation of TGF-β after lung irradiation can be reduced by co-administration of melatonin, potentially inhibiting the development of radiation-induced lung injury [38,39]. In light of all these studies, in our research, it was observed that OT suppressed TGF-beta levels and, in this context, prevented the fibrotic process in the lung.

The early discharge of VEGF leads to an elevation in the permeability of the pulmonary blood vessels, contributing to the development of lung edema and injury [40]. A Mura study found that a reduction in VEGF production and its receptor VEGF in lung tissues is linked to an elevated alveolar epithelial cell death rate, indicating that VEGF plays a protective role in cell survival [40]. Moreover, hypoxia induces the upregulation of VEGF and its corresponding receptors in pulmonary tissues, suggesting their involvement in the lung’s adaptive reaction to reduced oxygen levels. As observed from the blood gases, the VEGF increase observed after hypoxia and radiation therapy in the saline-treated group showed a dramatic decrease in the OT-treated group. Even on the 16th day of the application, significant improvement was achieved in the blood gas, histopathologic, and CT findings.

PDGF-related peptides in the alveolar airspace stimulate the migration and replication of mesenchymal cells, leading to the development of intra-alveolar fibrosis in patients with acute lung injury (ALI) [41]. This fibrosis process is a pathology that irreversibly reduces lung capacity in RILI and then leads to BOOP and then COPD. In this study, we slowed or stopped this process at the fibrosis stage with OT treatment after radiation therapy. 

We describe that the restorative molecules TGF-beta, VEGF, and PDGF, which are secreted to limit inflammation but cause fibrosis after radiotherapy, are reduced by OT administration. In contrast, bone morphogenic proteins-7 and prostacyclin increase after OT treatment. BMP-4 can regulate lung fibroblast function, while BMP-7 may specifically regulate TGF-1-induced profibrotic functions in lung fibroblasts, depending on the tissue or cell type [42]. In a study conducted by Yang et al., the authors found that BMP-7 effectively decreases silica-induced pulmonary fibrosis in rats, possibly by activating BMP/Smad signaling and inhibiting TGF-/Smad pathways [43]. On the other hand, BMP-7 is essential for developing alveoli in newborn rats with bronchopulmonary dysplasia. It controls the growth of lung fibroblasts by causing them to stop dividing during the G1 phase of the cell cycle [44]. However, in another study, contrary to our study, they suggested that the therapeutic potential of BMP-7 may be limited to the renal compartment, as it does not have an anti-fibrotic effect in lung or skin fibrosis [45]. In this study, the inhibition of TGF-beta (one of the most potent fibrotic indicator) by OT and the secondary increase in the level of BMP-7, which decreases the effect of TGF-beta, proved that OT suppresses the early fibrotic process in radiation pneumonitis.

Prostacyclin suppresses the growth and movement of smooth muscle cells in blood vessels, widens blood vessels, and prevents blood clotting in the lungs and the rest of the body [46]. Demling et al. suggested that infusing prostacyclin effectively safeguards the lung from injury caused by endotoxins, thereby decreasing pulmonary hypertension and vascular permeability [47]. An experimental study by Lovgren et al. discovered that targeting COX-2-derived prostacyclin could be a promising and innovative therapeutic strategy for treating pulmonary fibrosis in humans [48]. Moreover, Keith and colleagues declared that prostacyclin exhibits anti-inflammatory and anti-metastatic characteristics, effectively inhibiting the growth of tumors in lung cancer [49]. It is known that OT increases prostacyclin production [50]. In this study, prostacyclin production was augmented after OT administration, and this effect probably showed a fibrotic effect due to anti-inflammation. Decreasing pulmonary hypertension shows its effect by using blood and ingredients more effectively. 

This study was conducted in parallel with another study of OT administration in sepsis-induced acute lung injury [28]. The percentages of edema, fibrosis, and immune cell infiltration in morphometric measurements supported the strong histopathological evidence in this study. In this context, OT is again proven to be a potent anti-inflammatory agent.

## 5. Limitations

Since this study is an experimental animal study, more case series and meta-analyses are needed to make definite judgements on this subject.

## 6. Conclusions

In this study, biochemical, histological, and CT guidance has shown that serious but often overlooked radiation-induced lung damage—which can lead to long-term chronic lung damage secondary to radiotherapy and may present with findings mimicking viral pneumonia in the early period—can be reversed in the early period with OT treatment. Future studies will clarify the potential effect of OT on radiation-induced lung injury in acute and chronic periods.

## Figures and Tables

**Table 1:** Biochemical analysis of lung tissue results are presented as mean ± SEM.

	Normal Control Group Rats	RAD + Saline Group Rats	RAD + OT Group Rats
Lung TGF-beta level (pg/mg protein)	0.85 ± 0.04	1.46 ± 0.1 * p < 0.01	1.05 ± 0.06 # *p* < 0.05
Lung BMP-7 level (pg/mg protein)	0.45 ± 0.02	0.33 ± 0.08 ** p < 0.001	0.58 ± 0.03 ## *p* < 0.001
Lung VEGF level (pg/mg protein)	6.2 ± 0.4	12.5 ± 3.7 ** p < 0.001	8.9 ± 2.5 ## *p* < 0.001
Lung PDGF level (pg/mg protein)	0.38 ± 0.01	1.22 ± 0.09 * p < 0.01	0.71 ± 0.6 # *p* < 0.05
Lung Prostacyclin (pg/mg protein)	108.2 ± 15.1	64.5 ± 6.6 * p < 0.01	85.3 ± 7.05 # *p* < 0.05

Statistical analyses were performed using a one-way ANOVA test. * *p* < 0.01, ** *p* < 0.001 (different from control group), # *p* < 0.05, ## *p* < 0.001 (different from RAD and saline group).

**Table 2:** Morphometric analysis results are presented as mean ± SEM.

	Normal Control Group Rats	RAD + Saline Group Rats	RAD + OT Group Rats
Fibrosis percent (%)	1.5 ± 0.8	45.2 ± 6.5 * p < 0.0001	18.6 ± 4.9 # *p* < 0.001
Edema percent (%)	2.3 ± 1.1	32.1 ± 7.2 * p < 0.0001	10.7 ± 4.1 # *p* < 0.001
Immune cell infiltration percent (%)	6.4 ± 2.5	28.5 ± 5.4 * p < 0.0001	15.5 ± 7.8 # *p* < 0.001
CT [Hounsfield unit (HU) values]	618.5 ± 9.5	465.1 ± 8.8 * p < 0.0001	594.2 ± 10.7 # *p* < 0.001

Statistical analyses were performed using a one-way ANOVA test. * *p* < 0.0001 (different from control group), # *p* < 0.001 (different from RAD and saline group).

**Table 3:** Blood gas measurements results are presented as mean ± SEM.

	Normal Control Group Rats	RAD + Saline Group Rats	RAD + OT Group Rats
PaO_2_ (mmHg)	102.1 ± 8.2	90.2 ± 3.8 * p < 0.05	98.1 ± 6.5 # *p* < 0.05
PaCO_2_ (mmHg)	36.3 ± 2.9	51.1 ± 6.5 * p < 0.05	44.3 ± 5.9 # *p* < 0.05

A one-way ANOVA and a post hoc Bonferroni test were used to perform the statistical analyses. * *p* < 0.05, different from normal groups; # *p* < 0.05 different from FIP and saline group.

## Data Availability

All the data for this study are presented in the published article. Any further details are available from the corresponding author (Ahmet Kayalı, ahmet.kayali083@gmail.com) upon a reasonable request.

## References

[1] Kolilekas L., Costabel U., Tzouvelekis A., Tzilas V., Bouros D. (2019). Idiopathic interstitial pneumonia or idiopathic interstitial pneumonitis: What’s in a name?. Eur. Respir. J..

[2] Rahi M.S., Parekh J., Pednekar P., Parmar G., Abraham S., Nasir S., Subramaniyam R., Jeyashanmugaraja G.P., Gunasekaran K. (2021). Radiation-Induced Lung Injury-Current Perspectives and Management. Clin. Pract..

[3] Yan Y., Fu J., Kowalchuk R., Wright C., Zhang R., Li X., Xu Y. (2021). Exploration of radiation-induced lung injury, from mechanism to treatment: A narrative review. Transl. Lung Cancer Res..

[4] Liu X., Shao C., Fu J. (2021). Promising Biomarkers of Radiation-Induced Lung Injury: A Review. Biomedicines.

[5] Murofushi K.N., Oguchi M., Gosho M., Kozuka T., Sakurai H. (2015). Radiation-induced bronchiolitis obliterans organizing pneumonia (BOOP) syndrome in breast cancer patients is associated with age. Radiat. Oncol..

[6] Thomas R., Chen Y.H., Hatabu H., Mak R.H., Nishino M. (2020). Radiographic patterns of symptomatic radiation pneumonitis in lung cancer patients: Imaging predictors for clinical severity and outcome. Lung Cancer..

[7] Bledsoe T.J., Nath S.K., Decker R.H. (2017). Radiation Pneumonitis. Clin. Chest Med..

[8] Chen C., Zeng B., Xue D., Cao R., Liao S., Yang Y., Li Z., Kang M., Chen C., Xu B. (2022). Pirfenidone for the prevention of radiation-induced lung injury in patients with locally advanced oesophageal squamous cell carcinoma: A protocol for a randomised controlled trial. BMJ Open.

[9] Chen B., Na F., Yang H., Li R., Li M., Sun X., Hu B., Huang G., Lan J., Xu H. (2017). Ethyl pyruvate alleviates radiation-induced lung injury in mice. Biomed. Pharmacother. Biomed. Pharmacother..

[10] Hou G., Li J., Liu W., Wei J., Xin Y., Jiang X. (2022). Mesenchymal stem cells in radiation-induced lung injury: From mechanisms to therapeutic potential. Front. Cell Dev. Biol..

[11] İşeri S.Ö., Şener G., Saǧlam B., Gedik N., Ercan F., Yeǧen B.Ç. (2005). Oxytocin protects against sepsis-induced multiple organ damage: Role of neutrophils. J Surg Res..

[12] Donadon M.F., Martin-Santos R., Osorio F.L. (2018). The Associations Between Oxytocin and Trauma in Humans: A Systematic Review. Front. Pharmacol..

[13] Dusunceli F., İşeri S., Ercan F., Gedik N., Yeğen C., Yeğen B. (2008). Oxytocin alleviates hepatic ischemia-reperfusion injury in rats. Peptides.

[14] Houshmand F., Faghihi M., Zahediasl S. (2009). Biphasic protective effect of oxytocin on cardiac ischemia/reperfusion injury in anaesthetized rats. Peptides.

[15] Ondrejcakova M., Ravingerova T., Bakos J., Pancza D., Jezova D. (2009). Oxytocin exerts protective effects on in vitro myocardial injury induced by ischemia and reperfusion. Can. J. Physiol. Pharmacol..

[16] Tugtepe H., Şener G., Bıyıklı N.K., Yüksel M., Çetinel Ş., Gedik N., Yeğen B. (2007). The protective effect of oxytocin on renal ischemia/reperfusion injury in rats. Regul. Pept..

[17] Erkanli Senturk G., Erkanli K., Aydin U., Yucel D., Isiksacan N., Ercan F., Arbak S. (2013). The protective effect of oxytocin on ischemia/reperfusion injury in rat urinary bladder. Peptides.

[18] Gonzalez-Reyes A., Menaouar A., Yip D., Danalache B., Plante E., Noiseux N., Gutkowska J., Jankowski M. (2015). Molecular mechanisms underlying oxytocin-induced cardiomyocyte protection from simulated ischemiareperfusion. Mol. Cell. Endocrinol..

[19] Ryu S.H., Park E.Y., Kwak S., Heo S.H., Ryu J.W., Park J.H., Choi K.C., Lee S.W. (2016). Protective effect of α-lipoic acid against radiation-induced fibrosis in mice. Oncotarget.

[20] Bese N.S., Munzuroglu F., Uslu B., Arbak S., Yesiladali G., Sut N., Altug T., Ober A. (2007). Vitamin E protects against the development of radiation-induced pulmonary fibrosis in rats. Clin. Oncol. (R Coll Radiol.).

[21] Vujaskovic Z., Marks L.B., Anscher M.S. (2000). The physical parameters and molecular events associated with radiation-induced lung toxicity. Semin. Radiat. Oncol..

[22] Ao X., Zhao L., Davis M.A., Lubman D.M., Lawrence T.S., Kong F.M. (2009). Radiation produces differential changes in cytokine profiles in radiation lung fibrosis sensitive and resistant mice. J. Hematol. Oncol..

[23] Bradford M.M. (1976). A rapid and sensitive method for the quantitation of microgram quantities of protein utilizing the principle of protein-dye binding. Anal. Biochem..

[24] Davis S.D., Yankelevitz D.F., Henschke C.I. (1992). Radiation effects on the lung: Clinical features, pathology, and imaging findings. AJR Am. J. Roentgenol..

[25] Citrin D., Cotrim A.P., Hyodo F., Baum B.J., Krishna M.C., Mitchell J.B. (2010). Radioprotectors and mitigators of radiation-induced normal tissue injury. Oncologist.

[26] Yarnold J., Brotons M.C. (2010). Pathogenetic mechanisms in radiation fibrosis. Radiother. Oncol..

[27] Haddadi G.H., Rezaeyan A., Mosleh-Shirazi M.A., Hosseinzadeh M., Fardid R., Najafi M., Salajegheh A. (2017). Hesperidin as Radioprotector against Radiation-induced Lung Damage in Rat: A Histopathological Study. J. Med. Phys..

[28] Sever I., Ozkul B., Tanriover D., Ozkul O., Elgormus C., Gur S., Sogut I., Uyanikgil Y., Çetin E., Erbaş O. (2021). Protective effect of oxytocin through its anti-inflammatory and antioxidant role in a model of sepsis-induced acute lung injury: Demonstrated by CT and histological findings. Exp. Lung Res..

[29] Lin C., Tsai C., Chen T., Chang C., Yang H. (2019). Oxytocin maintains lung histological and functional integrity to confer protection in heat stroke. Sci. Rep..

[30] An X., Sun X., Hou Y., Yang X., Chen H., Zhang P., Wu J. (2019). Protective effect of oxytocin on LPS-induced acute lung injury in mice. Sci. Rep..

[31] Cui X., Zéni F., Vodovitz Y., Correa-de-Araujo R., Quezado M., Roberts A., Wahl S., Danner R., Banks S., Gerstenberger E. (2003). TGF-beta1 increases microbial clearance but worsens lung injury during Escherichia coli pneumonia in rats. Cytokine.

[32] Saito A., Horie M., Nagase T. (2018). TGF-β Signaling in Lung Health and Disease. Int. J. Mol. Sci..

[33] Wesselkamper S., Case L., Henning L., Borchers M., Tichelaar J., Mason J., Dragin N., Medvedovic M., Sartor M., Tomlinson C. (2005). Gene expression changes during the development of acute lung injury: Role of transforming growth factor beta. Am. J. Respir. Crit. Care Med..

[34] Ryu S.H., Lee S.W., Moon S.Y., Oh J.Y., Yang Y.J., Park J.H. (2010). Oncology, Establishment of a Radiation-induced Fibrosis Model in BALB/c Mice. Radiat. Oncol. J..

[35] Pittet J., Griffiths M., Geiser T., Kaminski N., Dalton S., Huang X., Brown L., Gotwals P., Koteliansky V., Matthay M. (2001). TGF-beta is a critical mediator of acute lung injury. J. Clin. Investig..

[36] Clodi M., Vila G., Geyeregger R., Riedl M., Stulnig T., Struck J., Luger T., Luger A. (2008). Oxytocin alleviates the neuroendocrine and cytokine response to bacterial endotoxin in healthy men. Am. J. Physiology. Endocrinol. Metab..

[37] Mittaud P., Labourdette G., Zingg H., Scala D. (2002). Neurons modulate oxytocin receptor expression in rat cultured astrocytes: Involvement of TGF-β and membrane components. Glia.

[38] Najafi M., Shirazi A., Motevaseli E., Geraily G., Amini P., Tooli L.F., Shabeeb D. (2019). Melatonin modulates regulation of NOX2 and NOX4 following irradiation in the lung. Curr. Clin. Pharmacol..

[39] Sheikholeslami S., Aryafar T., Abedi-Firouzjah R., Banaei A., Dorri-Giv M., Zamani H., Ataei G., Majdaeen M., Farhood B. (2021). The role of melatonin on radiation-induced pneumonitis and lung fibrosis: A systematic review. Life Sci..

[40] Mura M., Han B., Andrade C., Seth R., Hwang D., Waddell T., Keshavjee S., Liu M. (2006). The early responses of VEGF and its receptors during acute lung injury: Implication of VEGF in alveolar epithelial cell survival. Crit. Care.

[41] Snyder L., Hertz M., Peterson M., Harmon K., Marinelli W., Henke C., Greenheck J., Chen B., Bitterman P. (1991). Acute lung injury. Pathogenesis of intraalveolar fibrosis. J. Clin. Investig..

[42] Pégorier S., Campbell G., Kay A., Lloyd C. (2010). Bone Morphogenetic Protein (BMP)-4 and BMP-7 regulate differentially Transforming Growth Factor (TGF)-β1 in normal human lung fibroblasts (NHLF). Respir. Res..

[43] Yang G., Zhu Z., Wang Y., Gao A., Niu P., Tian L. (2013). Bone morphogenetic protein-7 inhibits silica-induced pulmonary fibrosis in rats. Toxicol. Lett..

[44] Sun Y., Fu J., Xue X., Yang H., Wu L. (2018). BMP7 regulates lung fibroblast proliferation in newborn rats with bronchopulmonary dysplasia. Mol. Med. Rep..

[45] Murray L., Hackett T., Warner S., Shaheen F., Argentieri R., Dudas P., Farrell F., Knight D. (2008). BMP-7 Does Not Protect against Bleomycin-Induced Lung or Skin Fibrosis. PLoS ONE.

[46] Kasza Z., Fetalvero K.M., Ding M., Wagner R.J., Acs K., Guzman A.K., Douville K.L., Powell R.J., Hwa J., Martin K.A. (2009). Novel signaling pathways promote a paracrine wave of prostacyclin-induced vascular smooth muscle differentiation. J. Mol. Cell. Cardiol..

[47] Demling R., Smith M., Gunther R., Gee M., Flynn J. (1981). The effect of prostacyclin infusion on endotoxin-induced lung injury. Surgery.

[48] Lovgren A., Jania L., Hartney J., Parsons K., Audoly L., FitzGerald G., Tilley S., Koller B. (2006). COX-2-derived prostacyclin protects against bleomycin-induced pulmonary fibrosis. American journal of physiology. Lung Cell. Mol. Physiol..

[49] Keith R., Geraci M. (2006). Prostacyclin in lung cancer. J. Thorac. Oncol..

[50] Williams K., Tahir K. (1980). Effects of uterine stimulant drugs on prostacyclin production by the pregnant rat myometrium. I. Oxytocin, bradykinin and PGF2 alpha. Prostaglandins.

